# Glioblastoma: Molecular Pathways, Stem Cells and Therapeutic Targets

**DOI:** 10.3390/cancers7020538

**Published:** 2015-03-25

**Authors:** Meena Jhanwar-Uniyal, Michael Labagnara, Marissa Friedman, Amanda Kwasnicki, Raj Murali

**Affiliations:** Department of Neurosurgery, New York Medical College, Valhalla, NY 10595, USA; E-Mails: m.labagnara@gmail.com (M.L.); marissa.d.friedman@gmail.com (M.F.); amandakwasnicki@gmail.com (A.K.); raj_murali@nymc.edu (R.M.)

**Keywords:** brain tumors, GBM, cancer stem cells, mTOR, therapies

## Abstract

Glioblastoma (GBM), a WHO-defined Grade IV astrocytoma, is the most common and aggressive CNS malignancy. Despite current treatment modalities, the survival time remains dismal. The main cause of mortality in patients with this disease is reoccurrence of the malignancy, which is attributed to treatment-resistant cancer stem cells within and surrounding the primary tumor. Inclusion of novel therapies, such as immuno- and DNA-based therapy, may provide better means of treating GBM. Furthermore, manipulation of recently discovered non-coding microRNAs, some of which regulate tumor growth through the development and maintenance of GBM stem cells, could provide new prospective therapies. Studies conducted by The Cancer Genome Atlas (TCGA) also demonstrate the role of molecular pathways, specifically the activated PI3K/AKT/mTOR pathway, in GBM tumorigenesis. Inhibition of the aforementioned pathway may provide a more direct and targeted method to GBM treatment. The combination of these treatment modalities may provide an innovative therapeutic approach for the management of GBM.

## 1. Introduction

Gliomas are tumors that arise from glial cells and are sub-classified as astrocytomas, glioblastomas, oligodendrogliomas, ependymomas, mixed gliomas, malignant gliomas not otherwise specified and other rare histological variants. Malignant gliomas are the most frequent and uniformly fatal cancers originating in the central nervous system. The World Health Organization (WHO) assigns four grades to astrocytomas: Grade I or pilocytic astrocytoma, Grade II or low-grade astrocytoma (AGII), Grade III or anaplastic astrocytoma (AGIII) and Grade IV or glioblastoma (GBM).

GBM is the most frequent and malignant histological type, accounting for 65% of gliomas. The incidence of GBM in the Unites States is 2.96 cases/100,000 population/year. GBM has a peak occurrence in adults older than 40 years of age with predominance in males. Despite the increased understanding of the oncological mechanisms underlying GBM pathophysiology and treatment advances, only an insignificant improvement in overall survival has been recorded during the first decade of the 21st century [1]. Although there are several current treatment modalities, including surgical resection, which, at times, is not possible, due to the vastly infiltrating growth of the glioblastoma cells, radiotherapy and chemotherapy, the median survival of patients diagnosed with GBM remains 12–15 months, and the five-year survival rate for GBM patients is less than 5% [2,3]. Invasion and relentless growth are considered the major causes of the meager therapeutic outcome [4]. All subtypes of high-grade neural tumors, including glioblastoma, anaplastic astrocytoma and anaplastic oligodendroglioma, share common malignant characteristics of preferential location in cerebral hemispheres, diffuse infiltration into normal brain parenchyma and unremitting tumor growth with fatal outcome within months or years.

Conventional GBMs constitute approximately 93% of all GBMs and can be divided into primary or secondary tumors: primary ones represent approximately 90% and develop *de novo*, whereas secondary ones arise from WHO-defined Grade II/III astrocytomas and represent about 5% to 10%. Histologically, both subtypes are indistinguishable. Primary GBM affects elderly individuals and is genetically characterized by EGFR amplification, PTEN mutation and p16INK4a deletion [5], whereas secondary GBM has TP53 and RB2 gene mutations and develops in patients with an average age of onset of 45. Loss of chromosome 10q is common in both subtypes of GBM [6]. The recently defined isocitrate dehydrogenase 1 gene (IDH1) is commonly mutated in secondary GBM [7]. Furthermore, selective mutations in the IDH1 gene are found in more than 70% of WHO Grades II and III astrocytomas and oligodendrogliomas, as well as in GBM that develop from lower-grade tumors [8]. In addition, often, tumors without mutations in IDH1 frequently display mutations of the IDH2 gene at the analogous amino acid site; and IDH1 mutations are associated with better survival [8]. Of note, it has been reported that secondary GBMs lacking IDH1 mutations have infrequent TP53 mutations, and patients with these tumors exhibit a shorter clinical history [9]. Moreover, a distinct progression history has been seen in GBM patients, as most secondary GBMs with IDH1 mutations progressed from a WHO Grade II, whereas secondary GBMs lacking IDH1 mutations developed through progression from anaplastic gliomas, WHO Grade III [9]. Comparative genomic analysis indicated that gains or the losses of various chromosomes carrying important oncogenes or tumor suppressor genes, respectively, including the gain of PIK3C2B, MDM4, KIT, PDGFRA, EGFR, GLI1, CDK4 and MDM2 and the loss of p16INK4a/p14ARF, PTEN and RB1, were common to all GBM tumors analyzed; whereas area-specific alterations included the gain of 14q32.33, where AKT1 is localized [10].

Genomic analysis categorized GBM into several subtypes: classical, mesenchymal, proneural and neural type [11,12,13]. These newly defined subtypes carry specific genetic anomalies, including discrete mutations of oncogenes or tumor suppressor genes, loss or gain of entire chromosomes or loss of a portion of a chromosome. The classical subtype is seen in about 21% of all GBMs. Amplification of EGFR can be considered the hallmark of this subtype, as it is seen in 97% of tumors. Loss of heterozygosity (LOH) of 10q23, harboring the PTEN locus, amplification of chromosome 7, loss of chromosome 10 and homozygous deletion at chromosome 9p21.3, encoding p16INK4A and p14 ARF, are also particularly common in this subtype. In addition, high expression of the Notch and Sonic hedgehog signaling pathways is also seen. Interestingly, the classical subtype of GBM tumors lack TP53 mutations. The mesenchymal subtype displays the expression of mesenchymal histologic markers, including; CHI3L1/YKL40, VEGF and CD44 [11,12,13]. Focal hemizygous deletion of 17q11.2, harboring the tumor suppressor gene NF1 (coding for neurofibromatosis-related protein NF-1 or neurofibromin 1), occurs predominantly in the mesenchymal subtype. Furthermore, mutations of NF1 are found in a larger proportion of GBM, accounting for 32% of core samples. The proneural subtype accounts for 31% of GBMs and has a high expression of oligodendrocytic genes, underlining its status as an atypical GBM subtype. The majority of TP53 mutations and TP53 LOH were found in proneural samples. Focal amplification of the locus at 4q12, harboring the PDGF receptor A (PDGFRA) gene, was seen in all subtypes of GBM, but at a much higher rate (35%) in proneural samples. Eleven of the twelve observed mutations in IDH1 are commonly found in this class and serve as diagnostic and prognostic markers [13]. Another group of tumors within the proneural subtype were found to express the glioma-CpG island methylator phenotype (G-CIMP). The 30% of proneural tumors expressing G-CIMP were associated with occurrence in younger patients with more favorable outcomes [14]. The classic GBM signature was less prevalent in this subtype and occurred in only 54% of these tumors. The neural subtype accounts for 16% of GBMs and is characterized by the expression of neuron markers. Chromosome 7 amplification associated with chromosome 10 loss is common in the neural subtype [13].

Recent studies have also attempted to classify GBM using immunohistochemical variations. Motomura *et al.* [15] analyzed 79 archival GBM samples using antibodies against 16 proteins selected according to The Cancer Genome Atlas (TCGA) classification (12,13) and identified four subcategories of GBM, namely the oligodendrocyte precursor (OPC) type, differentiated oligodendrocyte (DOC) type, astrocytic mesenchymal (AsMes) type and mixed type. Importantly, this histological classification confers the prognostic significance of GBM, where the OPC type with a positive IDH mutation shows a prolonged survival of 19.9 months [15]. Results from this study along with other genomic and proteomic analyses suggest the formulation of new guidelines for the WHO classification of central nervous system tumors, specifically GBM. Some of the proposed markers to be considered are mutations of IDH1, MGMT and 1p/19q co-deletion or ATRX loss, which carry significant diagnostic, prognostic and predictive abilities [16].

### 1.1. Cancer Stem Cells of GBM

Over the last decade, our understanding of biology has made it clear that stem cells not only have a critical role in the generation and maintenance of multicellular organisms, but are also involved in the development, growth and recurrence of tumors. Cancer stem cells (CSCs) carry three distinct properties: self-renewal, ability to differentiate into multiple lineages and extensive proliferative potential. The presence of CSCs was demonstrated in GBM through the identification of specific antigenic markers and the use of culture conditions that were originally developed for normal neural stem cells [17,18,19]. CNS cells grown *in vitro* form aggregates of cells, or free-floating neurospheres, which have the ability to differentiate into the various principle cell types of the brain (*i.e.*, neurons, astrocytes and oligodendrocytes). More importantly, these CSCs possess the ability to form new neurospheres, supporting their stem-like nature.

A subset of CSCs have been found to express a cell surface protein, CD133, a marker of hematopoietic and endothelial progenitors, that can be detected with an antibody specific for CD133. The purification of CD133+ cells from GBM tumors demonstrated this specificity and allowed for the growth and separation of a tumor stem-cell population. These tumor cells display stem cell-like properties, including neurosphere formation, self-renewal, high proliferative potential and multipotency. One study showed that as little as 100 CD133+ cells were capable of generating tumors with identical histopathological features of the parental tumors when grafted into NOD/SCID (non-obese diabetic, severe combined immunodeficient) mouse brains [19]. CD133+ cells were explicitly shown to form neurospheres with greater staining for proliferative markers as compared to CD133− cells [20]. In addition, clinical prognostic factors have also been shown to be associated with the CD133+ cell population, such that a higher expression of CD133+ cells correlates with a higher-grade malignancy [21]. On the contrary, another study showed that CD133− cells isolated from human GBM biopsies, which were then stereotactically implanted into mouse brains, resulted in tumor formation with both CD133+ and CD133− cells present. These findings suggest that CD133− cells also have the potential to initiate tumor formation [22]. Furthermore, populations of both CD133+ and CD133− tumor stem cells have the ability to form neurospheres, which are multipotent and capable of self-renewal without the influence of exogenous growth factors [23]. After researchers established that CD133+ cells are capable of forming GBM tumors, another study proposed the possibility that only certain subpopulations of CD133− cells have tumor initiating properties. The study subdivided CSC populations based on nestin, glial fibrillary acidic protein (GFAP) and neuron-specific enolase (NSE) expression. Researchers concluded that all CD133− cells were capable of tumorigenesis; however, there was a trend toward lower tumor formation rates for GFAP+ and NSE+ cells, suggesting that their tumorigenic ability may involve other molecular distinctions aside from CD133 expression [24]. Recently, another study has further elucidated the significance of CD133 expression in GBM tumor formation and aggressiveness. Researchers examined a specific subtype of glioma, a proneural tumor, which is initiated by PDGF-driven cells and characteristically expresses minimal amounts of CD133. The results demonstrated that both CD133+ and CD133− cells are capable of initiating proneural GBM tumors; however, CD133+ cells had a greater association with angiogenesis and, therefore, could lead to the formation of more aggressive tumor phenotypes [20].

In addition to CD133+, L1CAM is another molecular surface marker that has been found to be associated with more aggressive CSCs. Specifically, the areas of invasive fronts of the tumors expressed a particularly high amount of L1CAM [19]. L1CAM and CD133+ cells tend to cosegregate; however, L1CAM has not been used as a method to isolate CSCs [25]. Several other stem cell markers have been implicated in the increasing aggressiveness of glioblastoma tumors [26]. The use of these stem cell markers as serum biomarkers is still controversial and not widely accepted. Table 1 includes a list of the stem cell markers associated with GBM [27,28].

**Table 1:** Stem cell markers in GBM.

Stem Cell Markers	Type	Stem Cell Regulation
CD133	Surface Glycoprotein	Positivity associated with more aggressive tumors
L1CAM	Adhesion Molecule	Neuronal cell adhesion molecule required for maintaining the growth and survival of CD133-positive glioma cells with stem-like properties
CD44	Cell Surface Marker	Positivity associated with more aggressive tumors; localized with Id1 in the endothelial stem cell niche
A2B5	Surface Glycoside	Mixed evidence of association with more aggressive tumors
ID1	Transcriptional Regulator	Self-renewal
CD15 (aka-SSEA-1 or LeX)	Cell Surface Protein	CD15 is an enrichment marker of stem cells in CD133-negative tumors
Integrin α6	Transmembrane Receptor	Regulates self-renewal, proliferation and tumor formation by interacting with extracellular matrixes

**Figure 1:**
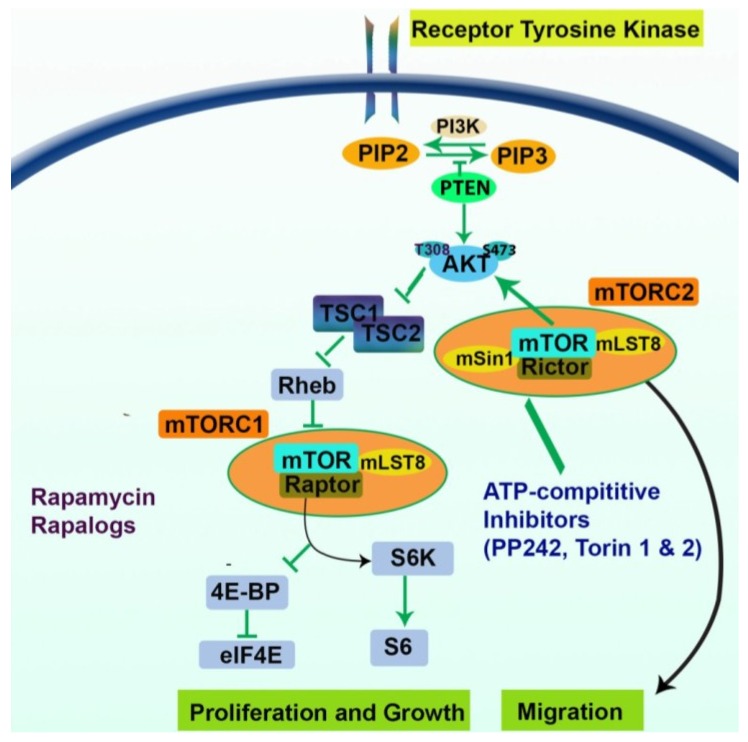
The PI3K/AKT/mTOR pathway in GBM. The inhibitors of this pathway may contribute to an anti-tumor effect in GBM. The diagram depicts two multiprotein complexes of mTOR, mTORC1 and mTORC2. Targeting both complexes may provide better treatment options, as the recently described small molecule inhibitors appear to be more effective than analogue binding inhibitors, such as rapamycin, which targets only mTORC1. This pathway is profoundly activated in GBM due to loss of tumor suppressor PTEN (see the text for details).

The aberrance of several development pathways has been implicated in the creation and maintenance of CSCs. One such pathway is PI3K, a family of the lipid/Akt/mTOR signaling cascade, which also involved regulation of brain functions [29]. Dysregulation of the PI3K/Akt/mTOR signaling pathway is evident in many types of cancers and may affect both tumorigenesis and therapy resistance. The downstream target mTOR plays a critical role in regulating protein synthesis, metabolism and angiogenesis. Dysregulation of mTOR is linked to the development of GBM, suggesting that inhibition of the mTOR pathway may have therapeutic value [30]. Recently, the role of mTOR signaling in the maintenance of GBM CSCs has been addressed [30,31]; however, the results remain controversial. Two major multiprotein complexes comprise mTOR (mTORC1 and mTORC2) and rapamycin, and its chemically-related compounds (also known as rapalogues) were used in clinical trials for the treatment of GBM. As shown in Figure 1, rapamycin inhibits only mTORC1 and not mTORC2; however, in recent years, several small molecules have been identified that directly inhibit mTOR by targeting the ATP-binding site; these include PP242, P30 and NVP-BEZ235. Two of these molecules, PP242 and P30, are the first potent, selective, ATP-competitive inhibitors of mTOR. As shown in Figure 1, unlike rapamycin, these molecules inhibit both mTORC1 and mTORC2 [31]. mTOR hyperactivation in both embryonic and adult stem cells led to the differentiation and depletion of the stem cell population. Furthermore, persistent activation of mTOR in normal epithelial stem cells results in exhaustion of these stem cells [32]. The mTOR pathway was also found to play a role in the senescence of several types of human and mouse cells [33,34]. Several studies have revealed that cellular stress, cytokines or activation of the mTOR pathway are able to increase the expression of CSC surface markers and phenotypes in certain bulk tumor cells [35,36,37,38], indicating that cell-extrinsic environmental factors may reprogram conventional tumor cells to cells with stem cell-like properties. Furthermore, different environmental conditions, such as hypoxia, have been shown to effect the malignant potential of CSCs. One mechanism of this is by activation of HIF-1α [39]. In human glioma cells, activation of HIF-1α, a positive downstream target of mTOR [40], enhances CD133+ glioma-derived cancer stem cell expansion by increasing self-renewal activity and inhibiting cell differentiation [41]. Consequently, the relationship between HIF-1α and CD133 expression remains to be established.

### 1.2. Therapeutic Implications of CSCs

**Figure 2:**
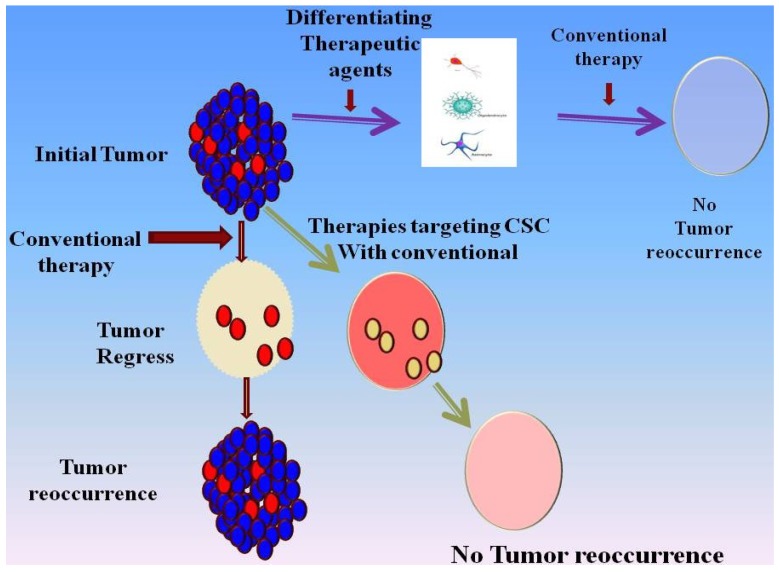
Figure describing the treatment options for tumors containing stem cell populations (see the text and [31,42] for details).

Reoccurrence of GBM is shown to be largely associated with the regeneration of tumor from remaining CSCs after initial treatment. Thus, targeting CSCs is an extremely important aspect of the clinical treatment of GBM. The functional aspects of CSC, such as cell proliferation and migration, are also important to consider, because they directly correlate with the invasive nature of GBM. One proposed mechanism for targeting CSCs is to first induce differentiation, thus making the cells more amenable to other therapeutic agents. A recent study by Friedman *et al.* examined this approach by illustrating that mTOR inhibition alone and in combination with differentiating agent, all-trans retinoic acid (ATRA), can target CSCs [42]. Such strategies are described in Figure 2. The results demonstrated that ATRA caused differentiation of CSCs, as evidenced by the loss of stem-cell marker nestin expression. Treatment of GBM cells with mTORC1 inhibitor rapamycin leads to nuclear localization of nestin. These observations were confirmed by Western blotting, which demonstrated a time-dependent decrease in nestin expression following ATRA treatment. Proliferation of CSCs, measured by neurosphere diameter, was decreased following treatments with ATRA alone and in combination with rapamycin. Of particular importance was the finding that the combined treatment of cells with mTOR inhibition and ATRA had a synergistic negative effect on CSC migration [42]. This synergism may be mediated by the MEK/ERK pathway given that treatment of cells with ATRA and MEK1/2 inhibitors resulted in the least amount of cell migration [42], perhaps due to their influence on differentiation. This is of particular interest, because resistance to the gold standard chemotherapeutic agent for GBM, temozolomide, was found to be mediated by MEK-ERK-induced activation of O(6)-methylguanine DNA methyltransferase (MGMT) [43]. One of the mechanisms of resistance against temozolomide is the high expression of the gene encoding O(6)-methylguanine DNA methyltransferase (MGMT), which removes the methyl group attached by temozolomide. A recent study demonstrated that MEK inhibition reduces MDM2 expression, which results in activation of p53, leading to p53-dependent downregulation of MGMT expression in CSC and, thereby, overcoming the temozolomide resistance. This further suggests that inclusion of MEK inhibitor with temozolomide treatment would make resistant GBM-CSC sensitive to temozolomide [43].

The effectiveness of inhibiting both ERK1/2 and mTOR was examined in other cancers. A phase I trial of 236 patients with advanced colorectal cancer treated with a PI3K inhibitor, a MAPK inhibitor or a combination of the two [44] showed that dual inhibition was superior in efficacy compared to inhibition of a single pathway alone. This may also provide an explanation for the only marginal benefits seen in an early phase trial of mTOR inhibitor (temsirolimus) as a sole treatment modality for recurrent GBM [45].

Other differentiating agents termed bone morphogenic proteins (BMPs), which are related to transforming growth factor-beta (TGF-β), were also shown to have therapeutic potential. BMP receptors 2 and 4 play a role in neuroepithelial proliferation and also induce differentiation of neurons and astrocytes. However, 20% of GBM tumors illustrate silencing of the active BMP target, BMPR1B, implying that its use as a therapeutic target may be limited to a specific population of patients [46,47,48,49].

### 1.3. MicroRNA

A newer and exciting approach to identifying and classifying GBM is through the use of miRNA. Forty three of 318 isolated miRNAs have been implicated in primary and recurrent GBM. miRNAs associated with GBM appear to interact with and regulate several molecular pathways, including p53, ErbB1, Notch, Wnt and TGF-β, thus implicating their dysregulation as a method of tumorigenesis [50]. miRNAs can either directly affect oncogenesis and tumor suppressive properties or manipulate various signaling pathways that modulate cancer growth, migration, stem cell regulation and therapeutic response. Furthermore, miRNAs alter the expression of phosphatase and tensin homolog (PTEN), platelet-derived growth factor (PDGF) and epidermal growth factor (EGFR), which are involved in the development and management of GBM. Huse *et al.* demonstrated that miR-26a influences the expression of tumor suppressor gene PTEN [51]. A subset of GBM is found to have upregulated levels of miR-26a and monoallelic PTEN loss. These observations suggest that miR-26a serves to silence residual PTEN transcript in PTEN^+/−^ tumors, which is analogous to the LOH event [51]. While the precise relationship between miR-26a and its role in PTEN expression is still being uncovered, a recent study suggested that oncogene c-myc controls the expression of PTEN via enhancing the levels of miR-26a [52]. In this manner, c-myc may activate the pAkt/mTOR signaling pathway, influencing cell survival and proliferation in GBM. In addition, suppression of miR-21 leads to a marked increase in PTEN levels [53]. Although increased levels of miR-21 enhance the development and proliferation of human GBM, an alternative study demonstrated that overexpression of oncogenic PDGF suppresses levels of miR-21 [54]. Suppression of miR-21 also downregulates the expression of the oncogenic EGFR pathway of GBM cells, independent of PTEN status [55]. Amplification of EGFR and miR-34 deletion were associated with shortened survival in GBM patients; these observations were also confirmed in patients from the TCGA project [12,56].

miRNAs have garnered interest for their ability to coordinate CSC stemness and differentiation. Enhanced expression of miR-128a, miR-504, miR-124a and miR-184 is correlated with a reduction of mesenchymal markers in GBM, implying that the presence of these miRNAs may predict a more favorable prognosis [57]. Similarly, overexpression of miR-21 reduced the expression of the neural stem cell marker, nestin, and enhanced the expression of astrocytic marker GFAP and neuronal marker TUJ1, suggesting its role in stem cell maintenance [58]. Conversely, inhibition of miR-221/222 enhanced nestin expression, while overexpression of these miRNAs is seen upon the differentiation of glioma-initiating stem cells [58]. miR-137 also inhibited GBM stem cell self-renewal and promotes differentiation by decreasing Oct4, Nanog and Sox2 levels [59]. Restoration of miR-153 in GBM stem cells also induced differentiation [60]. These findings highlight the involvement of miRNAs in the maintenance of CSCs and their prognostic implications in GBM (Table 2).

MicroRNAs are potential therapeutic targets, as they influence tumor cell growth, invasion, stem cell regulation, as well as chemoresistance. For example, restoration of miR-100 leads to suppression in tumor growth in GBM cells expressing reduced levels of this miRNA [61]. Researchers have also demonstrated reduced tumor growth with the overexpression of miR-211 and inhibition of miR-21 and miR-23 via MMP9 downregulation [62,63,64,65]. In particular, miR-218 inhibits glioblastoma invasion, migration, proliferation and stemness through various targets [54]. Furthermore, cellular migration and invasion of tumor cells is reduced with the inhibition of miR-10b [66]. Activation of the apoptotic pathway in GBM cells was achieved by overexpression of miR-211 [62]. Manipulation of miRNAs can also be combined with GBM chemo-radiation therapies. Standard GBM therapy includes the use of a chemotherapeutic alkylating agent, temozolomide (TMZ). Drug resistance and elevated expression of miR-21 occurs with chronic use of TMZ, whereas treatment with miR-21 inhibitors along with TMZ treatment causes significant apoptosis or cell death compared to TMZ treatment alone [67]. Similarly, knockdown of miR-195 significantly enhances apoptosis when given in combination with TMZ, whereas suppression of miR-455-3p or miR-10a displays a moderate cellular killing effect in combination with the drug [68]. Notably, Costa *et al.* demonstrated that silencing miR-21 also enhanced the effect of tyrosine kinase inhibitor sunitinib [53]. Aside from their role as an adjuvant to chemotherapy, miRNAs also mediate responsiveness to radiation therapy. Overexpression of miR-26a downregulates the DNA repair protein, ATM, causing inhibition of homologous recombination repair and sensitizing GBM cells to radiotherapy [69]. Studies have also shown that combination chemo-radiation therapy reduces miR-21 expression, and thus, miR-21 may be a potential marker of therapy responsiveness [70].

**Table 2:** MicroRNA in regulation of GBM stem cells and other functions. TMZ, temozolomide.

MicroRNA	Functions
**Stem Cell Regulation/Migration, Invasion, Apoptosis**	
miR-26a upregulation	Monoallelic PTEN loss
miR-21 suppression	Increases levels of PTEN
	Down-regulates EGFR expression
PDGF overexpression	miR-21 suppression
EGFR amplification	Shortens survival in GBM patients
miR-34 deletion	
miR-128a, miR-504, miR-124a or miR-184 enhanced expression	Reduces levels of mesenchymal markers in GBM
miR-21 overexpression	Decreases nestin expression
	Enhances GFAP and TUJ1 expression
miR-221/222 inhibition	Enhances nestin expression
miR-137 expression	Inhibits GBM self-renewal
	Decreases Oct4, Nanog and Sox2 expression
Restoration of miR-153	Induces GBM stem cells differentiation
miR-211 overexpression	Suppression of tumor growth
miR-21 and miR-23 inhibition	
miR-100 restoration	
miR-218	Inhibits glioblastoma invasion, migration, proliferation and stemness
miR-10b inhibition	Reduces cell migration and invasion
miR-211 overexpression	Activates apoptotic pathway
**Therapy**	
Chronic use of TMZ	Elevates miR-21expression
TMZ + miR-21 inhibitors	Significant apoptosis and cell death
miR-195 knockdown + TMZ	Enhances apoptosis
miR-455-3p or miR10a suppression + TMZ	Induces moderate cellular killing
miR-21 silencing	Enhances effect of Sunitinib
miR-26a overexpression	Downregulates ATM
	Sensitizes GBM cells to radiotherapy

### 1.4. Gene Therapy

The ideal treatment for any malignancy, including GBM, is induction of tumor cell-specific cytotoxicity without affecting normal cells. Therefore, systemic chemotherapy for brain tumors is not ideal. A recent review article discusses various gene therapies that have been explored as therapeutic approaches for patients with GBM [71]. In addition, even localized radiotherapy does not spare the normal cells in the tumor bed. Localized chemotherapy in the form of Gliadel, carmustine (bis-chloroethylnitrosourea; BCNU) wafers left in the resection bed at the time of surgery have shown efficacy in treating GBM with a slightly higher rate of wound healing complications, but its effects are not tumor cell specific [72]. This is the only approved local intra-cavity therapy. Previous attempts to use a viral vector to transfect tumor cells using suicide gene therapy were attempted with mild success. Recently, the results of an open label phase III trial [73] investigating a locally administered gene therapy were published and are reviewed below.

A replication-deficient adenovirus vector, cDNA coding for HSV-tk (herpes simplex virus thymidine kinase), was administered into the resection bed of patients with GBM. This enzyme phosphorylates ganciclovir to ganciclovir triphosphate, a cytotoxic analogue, which selectively kills dividing cells via incorporation into DNA, leading to apoptosis of the transfected cells, as well as the neighboring tumor cells [74,75,76]. A five-day period was allowed for transduction, and from Days 5–19 postoperatively, the patients were given ganciclovir intravenously twice daily. This process is believed to spare normal neurons, because they do not proliferate and are therefore not susceptible to the toxic metabolites [77,78]. Patients from 38 sites in nine countries were randomized into two groups, and 119 patients were placed into the treatment group with 117 patients in the non-treatment group. The treatment group was given 30–70 local injections of 100-microliter aliquots up to a depth of 2 cm in the walls of the resection cavity with a blunt needle. These patients were infused with ganciclovir as described above. Both groups varied with respect to “standard of care” in that not all sites had temozolomide available, and a small group of patients in both groups did not receive radiotherapy. The primary endpoint of the study was whether this treatment increased the time to death or reintervention and if this goal was achieved. However, the intervention failed to improve overall survival. The improvement in median time to death or reintervention was irrespective of temozolomide usage, which was statistically significant. Eighty-eight patients (71%) in the experimental group had one or more treatment-related adverse events, compared with 51 (43%) patients in the control group. Most adverse events were mild and self-limited, such as seizures or brain inflammation. The most common serious adverse events were hemiparesis (eight in the experimental group, three in the control group) and aphasia (six and two, respectively).

The theory and methodology behind this study is sound, but unfortunately, the results show that there is a marginal benefit with moderate risk. Further investigation is needed to optimize delivery and to decrease the rate of adverse events before gene therapy can become incorporated into the standard of care for GBM.

### 1.5. Immunotherapy in Treatment of Glioblastoma

Multiple phase I, II and III clinical trials investigating the use of cancer immune-vaccine therapy for patients with GBM are currently ongoing. Detailed information can be found at “Clinicaltrials.gov”, and reviewed recently by Galluzzi *et al.* 2012, and Aranda *et al.* 2013 [79,80]. The cancer vaccine hypothesis is based on the foundation that vaccinating GBM patients with their own dendritic cells, which are programmed against their tumor or allergenic cancer stem cells, can provide immunity against residual cancer cells that may be present despite prior treatment. The ultimate goal of such vaccine studies is to form immune system responses against a patient’s own cancer cells. GBM patients’ own immune-stimulating dendritic cells are isolated via leukapheresis. Further, the dendritic cells are customized to generate an immune response against the tumor antigen or cancer stem cells by combining them and allowing the dendritic cells to mature. The resulting product is then vaccinated. Dendritic cell vaccines for patients with malignant glioma demonstrated improvement in median survival and five-year survival in newly diagnosed, as well as recurrent Grade IV gliomas, compared to historical controls [81].

An investigation therapy, known as DCVav^®^-L, is currently being investigated in newly diagnosed GBM patients who undergo surgery. After two phase I/II trials, a large phase III trial is currently ongoing [82]. Trials consisted of 39 patients, including 20 patients with newly diagnosed GBM and 19 patients with recurrent GBM and other gliomas. Patients were given trimodal therapy, including radiation and temozolomide (Temodar). The procedure for this experimental therapy involved extracting patients’ tumor lysate and then mixing with the patients’ DCs to make them recognize GBM cells (antigens). These immune-modified DCs were then given to GBM patients. The main end-point was to evaluate disease progression-free survival (PFS) and overall survival time following treatment with DCVax(R)-L. The response to these vaccines is correlated with the body’s natural immune system to target neoplastic cells. Another trial demonstrated that relapsed GBM patients treated with DC vaccine-based therapy demonstrate longer survival benefits with faster vaccine schedules [83]. The multicenter clinical trial of DCVax showed some encouraging results. The newly diagnosed patients who received DCVax in addition to standard of care treatment typically did not have tumor recurrence for a median of approximately two years (more than triple the usual time with standard of care treatments), and the median survival was approximately three years (about 2.5-times the usual period of survival with standard of care treatment) [84].

In recent years, researchers have analyzed peripheral blood lymphocyte population (PBL) for immunoregulatory factors and have suggested their role as predictive prognostic markers in patients receiving the DC vaccine. However, the efficacy of this vaccine is dependent on the presence of negative costimulatory molecules, such as CTLA-4 and PD-1. Researchers have demonstrated that decreasing the expression of CTLA-4 in peripheral blood T-cell lymphocytes of GBM patients improves overall survival following treatment with the DC vaccine [85].

Interestingly, a number of other immunotherapeutic approaches to target EGFRvIII, a unique deletion mutant of EGFR, have been explored. Given the technical difficulty and relatively high cost of dendritic cell vaccine therapy, targeting EGFRvIII appears to be a better option, as shown in both murine tumor models and early clinical trials. Researchers have developed a peptide-based approach to target EGFRvIII. PEPvIII, derived from the novel fusion junction amino acid sequence (H-Leu-Glu-Glu-Lys-Lys-Gln-Asn-Tyr-Val-Val-Thr-Asp-His-Cys-OH) [86], is a well-characterized, EGFRvIII-specific, 14-mer peptide that has been shown, when coupled to keyhole limpet hemocyanin (KLH), to elicit both humoral and cellular immune responses. Clinical trials using this therapeutic vaccine are currently underway [87].

## 2. Conclusions

In this review, we summarized recent developments surrounding the treatment of GBM. We focused on molecular pathways commonly used to identify CSCs of GBM related to self-renewal, differentiation and therapy resistance. We also presented new immunotherapy, which is currently under phase III clinical trials to improve the survival of patients with GBM. Moreover, we identified miRNAs as potential therapeutic targets, as they influence tumor cell growth, invasion, stem cell regulation, as well as chemoresistance in GBM. Despite the complex genetic composition of this malignancy, several approaches are aimed at understanding the biology of the disease, as well as identifying various ambiguous markers, which may provide effective novel therapies against GBM.
